# Mechanisms for the Invasion and Dissemination of *Salmonella*

**DOI:** 10.1155/2022/2655801

**Published:** 2022-06-09

**Authors:** Qiao Li

**Affiliations:** Tongji Hospital, Tongji Medical College, Huazhong University of Sciences and Technology, Wuhan, Hubei, China

## Abstract

*Salmonella enterica* is a gastroenteric Gram-negative bacterium that can infect both humans and animals and causes millions of illnesses per year around the world. *Salmonella* infections usually occur after the consumption of contaminated food or water. Infections with *Salmonella* species can cause diseases ranging from enterocolitis to typhoid fever. *Salmonella* has developed multiple strategies to invade and establish a systemic infection in the host. Different cell types, including epithelial cells, macrophages, dendritic cells, and M cells, are important in the infection process of *Salmonella*. Dissemination throughout the body and colonization of remote organs are hallmarks of *Salmonella* infection. There are several routes for the dissemination of *Salmonella* typhimurium. This review summarizes the current understanding of the infection mechanisms of *Salmonella*. Additionally, different routes of *Salmonella* infection will be discussed. In this review, the strategies used by *Salmonella* enterica to establish persistent infection will be discussed. Understanding both the bacterial and host factors leading to the successful colonization of *Salmonella enterica* may enable the rational design of effective therapeutic strategies.

## 1. Introduction

*Salmonella* is a Gram-negative, intracellular pathogen. There are more than 2,600 serovars of *Salmonella* characterized to date that are differentiated on the basis of the lipopolysaccharide (LPS) O antigen and the flagellar H antigen [1]. There are just two species of *Salmonella*: *S. enterica* and *S. bongori* [2]. *Salmonella* typhi and *Salmonella* typhimurium are well-known members of the *S. enterica* species. *S.* typhimurium and *S. enteritidis* are predominantly associated with gastroenteritis in humans [3]. Each year, there are about 155,000 deaths due to nontyphoidal *Salmonella* (NTS) infections. *Salmonella enterica* serovar typhi infections cause a staggering 20 million infections and 200,000 deaths annually [4]. Gastroenteritis induced by Salmonella infections is a major cause of morbidity and mortality in children under 5 years of age [5]. Diarrhea caused by *Salmonella* species causes a global human health burden that contributes to significant annual morbidity and mortality and requires new therapeutic strategies for effective management. Almost 60% of *Salmonella* strains have developed resistance to first-line antibiotics [6]. Most patients recover from infections after treatment. However, 3–5% of patients become chronic carriers, with chronic infection in the gall bladder [7]. Chronic carriers can intermittently shed the bacteria through their feces and urine throughout the rest of their lives [8]. The liver is also a reservoir for chronic infections with *Salmonella* Typhi; from the liver, the bacteria can be intermittently shed into the gallbladder [9].

*Salmonella* typhi infections can cause fever, hepatomegaly, splenomegaly, and bacteremia. In the disease process, the bacteria disseminate into the gall bladder, liver, and spleen [10]. Approximately 90% of chronic *Salmonella* carriers have gallstones [10–12], and are at significantly increased risk for gallbladder cancer (GC) [12, 13]. DelGiorno et al. reported that persistent *Salmonella* infections can cause pancreatitis in a murine model of infection [14]. Some *Salmonella* carriers are asymptomatic. Roughly 2–5% of *Salmonella*-infected patients fail to clear the bacteria within one year [12]. Such chronic infections, especially asymptomatic infections, pose a huge socioeconomic burden, especially in South Asian and African countries, by unknowingly spreading infections to others, who may experience symptomatic infections and suffer economic costs as a result. Understanding the cellular routes of *Salmonella* invasion and dissemination in the host and the mechanisms of *Salmonella* persistent infection may facilitate the exploration of novel treatment strategies for patients with chronic infections. Ultimately, this may help eliminate the asymptomatic carriage of *Salmonella* as a concern for public health.

*Salmonella* infections can result from the ingestion of contaminated foods because they can survive the low pH of the stomach [15]. Although bile in the small intestine poses a challenge for *S.* typhimurium, the PhoQ/PhoP two-component regulatory system mediates resistance to bile [16]. *Salmonella* predominantly causes inflammation of the terminal ileum and colon [17]. *S*. typhimurium can spread systemically in mice, and *S.* typhimurium infections in mice are used as an animal model for typhoid fever in humans [18]. Pretreatment of mice with streptomycin prior to*Salmonella* infection disturbs the healthy microbiota and facilitates infection of the intestinal lumen with *Salmonella* typhimurium [19]. Streptomycin-treated mice are therefore often used as animal models of *S.* Typhimurium-induced gastroenteritis [19]. Before the oral infection of *S*. typhimurium and *S*. enteritidis, approximately 20 mg of streptomycin treatment by intragastric administration in the mice will allow a high colonization level in the cecum and colon of the mice [20]. Acute microbiota depletion will reduce the colonization resistance and facilitate the infection of the bacteria. Microbiota can limit *Salmonella* colonization, and diet can affect microbiota composition. Low-fiber or high-fat diets will increase *S*. typhimurium colonization in mice [21]. Fat can promote *S*. typhimurium infection in mice by eliciting bile salts, which help fat digestion [21]. A high-fat diet will cause microbiota perturbation [21]. *E. coli* may limit *S*. typhimurium infections during diet shifts [21].

Mice with a mutation in the natural resistance-associated macrophage protein 1 gene (Nramp+), such as CL57/BL6 or BALB/C mice, are susceptible to *Salmonella* infection [22]. Nramp1 is a macrophage-specific exporter, and the Nramp1 gene codes for an ion transporter that pumps ions out of *Salmonella*-containing vacuoles (SCV) [22]. The SCV is the intracellular vacuolar niche in which *Salmonella* can replicate and achieve dormant infection. wild type 129 × 1/Sv mice, which possess the Nramp^+/+^ allele, are used as an animal model for chronic *S*. typhimurium infection [23]. Mice with a mutation in the natural resistance-associated macrophage protein 1 gene (*Nramp1*), such as CL57/BL6 or BALB/C mice, are susceptible to *Salmonella* infection [22]. Nramp1 is a macrophage-specific exporter, and the *Nramp1* gene codes for an ion transporter that pumps ions out of SCV [22]. The SCV is the intracellular vacuolar niche in which *Salmonella* can replicate and achieve dormant infection. Wild type 129 × 1/Sv mice, which possess the *Nramp1*^*+/+*^ genotype, are used as an animal model for chronic *S*. typhimurium [23].

## 2. M Cells

**Figure 1:**
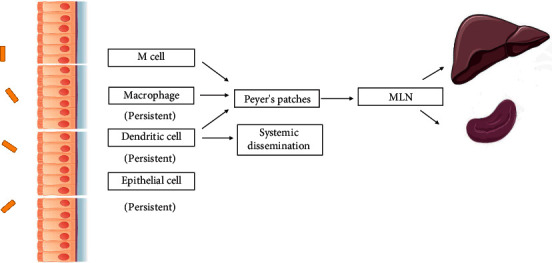
The multiple routes of *Salmonella* dissemination. *Salmonella* can be phagocytized by macrophages or dendritic cells and disseminate to the Peyer's patches (PP), mesenteric lymph nodes, and eventually the liver and spleen. *Salmonella* can also reach the circulation by the transportation of dendritic cells, e.g., CD18-expressing phagocytes. *Salmonella* can also invade the intestinal epithelial cells directly by way of a zipper or trigger mechanism. Salmonella can achieve persistent infection in epithelial cells and phagocytic cells. The dormant persisters can be released and induce the recurrence of the infection.

Enteropathogenic infections start in the intestinal lumen. Dissemination through microfold or membranous (M) cells is one of the best-understood routes of *Salmonella* dissemination [24]. M cells are specialized follicle-associated epithelial (FAE) enterocytes on the surface of mucosa-associated lymphoid tissues [25, 26]. *Salmonella* typhimurium initiates infection in mice by infecting and destroying the specialized epithelial M cells and then traveling to the mesenteric lymph nodes [24]. See Figure 1.

*Salmonella* directly invades M cells but can also transform follicle-associated epithelial cells into M cells to provide additional routes for intestinal invasion [27]. Indeed, Tahoun et al. found that *S*. Typhimurium can induce an epithelial-mesenchymal transition (EMT) of FAE enterocytes and transition the FAE to M cells [27]. These processes rely on the bacterial type III effector protein SopB [27]. Through the activation of NF-*κ*B and Wnt/b-Catenin signaling pathways, *Salmonella* induces host cell transdifferentiation through receptor activator of NF-kB ligand (RANKL) [27]. This finding was the first report that *S*. typhimurium can transform epithelial cells into M cells using a single virulence factor.

Intestinal immunity is the first defense barrier that enteropathogens encounter during infection. Lymphotoxin signaling is important for maintaining intestinal immune balance. LT*β*R can also be activated by lymphotoxin (LT*αβ*) [28]. Lymphotoxin signaling promotes the differentiation of M cells from intestinal epithelial cells [29]. This signaling is involved in the regulation of intestinal inflammation, as shown by the DSS-induced colitis model [30]. Mice with knocked-out lymphotoxin signaling molecules (LT*α*_3_, LT*α*_2_*β*_1_, and LT*α*_1_*β*_2_) have abnormal lymphoid development [31]. Lymphotoxin *β*-receptor knockout mice lack all lymph nodes and gut-associated lymphatic tissues, including Peyer's patches (PPs) [32]. These lymph node-defective mice are a good model for the systemic dissemination of *S*. typhimurium. Infection of *Salmonella* in LT*β*R^−/−^ mice demonstrates that organized lymph tissues are dispensable for the systemic infection of the host [20]. As shown by a study from Barthel et al., without Peyer's patches (PPs), bacteria can still reach remote organs [20]. This phenomenon indicates the importance of dendritic cell-mediated transportation in the dissemination of *S*. typhimurium [33]. *Salmonella* exploited dendritic cells as vesicles for dissemination. Cheminay et al. showed that after infection by *Salmonella*, dendritic cells could upregulate the CCR7 receptor and migrate via the CCR7 ligands CCL19 and CCL211 [13, 33–35].

A study by Wroblewska et al. showed that lymphotoxin signaling is essential for the clearance of *Salmonella* from the intestinal lumen [36]. A lack of LT*β*R signaling did not impact the initiation of inflammation induced by Salmonella. However, the resolution of Salmonella infection was impaired [36]. The infectious processes in *S*. typhimurium in WT and LT*β*R^−/−^ mice lacking Peyer's patches (PPs) and MLN are highly similar [20].

## 3. Epithelial Cell

*S*. typhimurium can invade polarized gallbladder epithelial cells and replicate inside the epithelial cells [37]. Gallbladder epithelial cells are a reservoir for *Salmonella* colonization [37]. Long-term colonization of *Salmonella* in the gallbladder cells can drive the premalignant transformations of the cells. *Salmonella* can invade the polarized gallbladder cells. *Salmonella* can induce the extrusion of epithelial cells, which is accompanied by caspase-1 activation-related cell death. Epithelial cells can provide a shelter for the bacteria to survive and replicate in the cytosol of the epithelial cells [38, 39]. The type III secretion system is involved in the priming of the bacteria for invasion. Cytosolic bacteria can induce the extrusion of epithelial cells and be released into the intestinal lumen [38, 39].

Unlike M cells, *Salmonella*'s invasion of epithelial cells does not rely on phagocytosis. The type III secretion system (T3SS) is the most important virulence factor for *Salmonella* species, and one is encoded on *Salmonella* pathogenicity island 1 (SPI1) and the other is encoded on *Salmonella* pathogenicity island 2 (SPI2) [40]. The type III secretion system is a molecular syringe that can translocate the effector proteins directly from the bacteria into the cytosol of cells. Effector proteins are injected into the cytoplasm of the host by a T3SS gene cluster. SPI1 is involved in the invasion process of *Salmonella* [41]. After invading host cells, *Salmonella* survives in SCVs by using elements encoded on SPI2 [42–45]. Approximately 4–6 h after the cellular invasion, bacterial replication is initiated [46].

*Salmonella* can induce membrane ruffling in intestinal cells to cause them to engulf the bacteria [47]. Various *S*. Typhimurium fimbrial operons contribute to bacterial attachment and invasion of epithelial cells [48]. The zipper and trigger mechanisms are two well-studied mechanisms of *Salmonella* entry into epithelial cells [49, 50]. The trigger mechanism is activated by the type III secretory system [49]. SipB/C in *Salmonella* type III secretory system assembles a pore in the epithelial cell, bacteria and epithelial cells can contact through the continuum created by the SipB/C [49]. Cytoskeletal reorganizations known as “membrane ruffles” and “internalization” are two key elements of the trigger mechanism [51]. Bacteria are internalized in SCV following a trigger mechanism [49].

In contrast, there are only minor cytoskeletal protein rearrangements involved in the zipper mechanism [50]. Instead, the zipper mechanism is mainly mediated by interactions between bacterial ligands such as Rck and host cell surface receptors [52]. There are many outer membrane proteins that participate in the invasion process of *Salmonella* typhimurium [53]. Rck is a 17 kDa outer membrane protein (OMP), which are membrane proteins found in the outer membranes of Gram-negative bacteria. Rck is encoded by the rck gene on the large virulence plasmid [54]. They are a family of highly conserved OMPs within the Enterobacteriaceae family. This receptor binding leads to downstream signal activation mediated by the phosphorylation of tyrosine kinase. The zipper mechanism is activated by the binding of host cell receptors by the bacterial ligands. Actin polymerization and membrane extension are initiated by the activated downstream signaling.

PagN is another OMP [54] and is widely conserved in the *Salmonella* genus [55]. The PagN protein interacts with cell surface heparin sulfate proteoglycans to invade cells [53]. Binding between OmpV and the extracellular matrix components fibronectin and *α*1*β*1 integrin leads to the adhesion of *Salmonella* typhimurium to intestinal epithelial cells and ultimately activates actin modulation [56]. PAMPs of Salmonella can be recognized by the innate immune response receptors through MyD88-dependent TLR signaling [57]. Infection with SPI1 T3SS disrupted Salmonella can still induce colitis in C57BL/6 mice through a mechanism that is dependent on MyD88 signaling [58]. The effectors of type III secretion systems in the invasion and dissemination of *Salmonella* are summarized in Table 1.

The binding of pattern recognition receptors (PRRs) with pathogen-associated molecular patterns (PAMPs), including peptidoglycan, lipopolysaccharide, flagellin, can mediate *Salmonella* invasion [64, 65]. TLR4 and TLR5 play a role in the host response to *Salmonella* [66]. In human macrophages, *Salmonella* can activate NAIP/NLRC4 and canonical NLRP3 Inflammasomes by its flagellin [67]. Caspase-1 will be activated after binding with NLRC4 and NLRP3 inflammasomes in response to *Salmonella*. *Salmonella* colonization was much higher in caspase 11 deficient mice than in wild-type mice [68]. Casp1^−/−^ and Casp1/11^−/−^ monolayers showed significantly increased intracellular bacteria, accompanied by low intestinal epithelial cells (IECs) shedding and death [68]. Caspase activation is important for limiting the intracellular replication of *Salmonella* [68].

## 4. Dendritic Cells

Intestinal dendritic cells are found in Peyer's patches [69], in the lamina propria [70], in the subepithelial dome [71], and under the follicle epithelium [72, 73]. The phagocytosis of *Salmonella* by dendritic cells and macrophages is mediated by the interactions between specific pathogen-associated molecular patterns (PAMP) and cellular receptors on the phagocyte surface, such as pattern recognition receptors (PRRs), which include Toll-like receptors (TLRs), NOD-like receptors (NLRs), and C-type Lectin receptors [74]. NOD-like receptors (NLRs), nucleotide-binding leucine-rich repeat-containing proteins, are intracellular innate immune receptors that belong to the pattern recognition receptors (PRRs) [75]. NLR is short for nucleotide-binding domain leucine-rich repeat. MyD88-and TRIF-dependent pathways can be regulated by NLRs [75].

Dendritic cells are exploited by *Salmonella* typhimurium as “Trojan horses” to enable systemic dissemination [76]. This strategy of manipulating host cell migration to facilitate broader dissemination is common among other pathogens such as *Mycobacterium tuberculosis*, HIV, and a range of other Gram-negative bacteria [77–80]. For example, after phagocytes are infected by HIV, the gp120 protein on the virus binds with the C-type Lectin receptor DC-Sign, initiating phagocytosis by dendritic cells that then migrate to lymph nodes and release viral particles that proceed to infect CD4+ lymphocytes [77–79]. Several Gram-negative bacteria also disseminate through antigen-presenting cells, as demonstrated by Yang et al. [76, 81–88]. After binding to C-type lectin receptors with core LPS, bacteria are transported throughout the host by antigen-presenting cells [76, 81–88].

By exploiting migratory dendritic cells, the *Salmonella* can thus traffic from the intestinal lumen to systemic organs [34]. During active infection, the dendritic cells' expression of CCR7, a receptor for the chemokines CCL19 and CCL21, is increased [34]. This allows dendritic cells to migrate along chemotactic gradients to remote sites like the lymph nodes and spleen [34]. *Salmonella* survives inside the dendritic cells, subverts the function of dendritic cells, impairs the activation of adaptive immune responses, prevents fusion and lyso-endosomal degradation, and achieves systemic dissemination [45]. Cheminay et al. published the first example that *Salmonella* can inhibit antigen presentation by dendritic cells by altering MHC-II-dependent antigen presentation in an SPI2-dependent manner [89]. Through subversion of the antigen presentation of dendritic cells, the bacteria reduce the activation of the active immune response. Lapaque et al. demonstrated that *Salmonella* can inhibit the surface expression of MHC class II antigens on dendritic cells through ubiquitination [90].

CD103^+^CD11b^+^ DCs have been reported to transport *Salmonella* typhimurium to the mesenteric lymph nodes (MLN) after oral infection [91]. CD103^+^ dendritic cells (DCs) typically phagocytose bacteria from the small intestine and present antigens to T cells [91]. Another group of dendritic cells that can facilitate the dissemination of *Salmonella* is intestinal CD11c^+^ lamina propria cells (LPCs), which do so in a TLR5^−^dependent manner [92]; the migration of *Salmonella* typhimurium from the intestinal tract to MLN is impaired in TLR5^−/−^ mice. In TLR5^−/−^mice, migration of bacteria by CD11c + LPCs is impaired [92, 93].

Distinct populations of dendritic cells participate in the processing and immune sampling of *Salmonella*. Specialized DC subsets in Peyer's patches (PPs), CCR6 (+) DCs, are recruited to the dome regions of Peyer's patches (PPs) to sample the bacteria and present to CD4^+^ T cells [94, 95]. CX3CR1-positive lamina propria DCs take up *S*. typhimurium by transepithelial processes [96]. Indeed, CX3CR1 deficiency leads to reduced bacterial sampling in the intestinal lumen by lamina propria DCs [96]. Further, these CX3CR1-positive DCs lacked CCR6 expression, which is different from the Peyer's patches (PPs) associated-dendritic cells [96].

*S*. typhimurium can be taken up by sub-epithelial DCs and can survive within murine PP dendritic cells [97]. The *S*. typhimurium strain PhoP^c^ has a point mutation in the phoP/Q locus [98] that attenuates its capacity to survive in macrophages but was able to persist for several weeks *in vivo* [97]. *Salmonella* can persist in the dendritic cells in the Peyer's patch. They can also be directly sampled by dendritic cells that express tight junction proteins, such as the interepithelial dendritic cells in the intestinal villi that penetrate gut epithelial monolayers by opening tight junctions and directly sampling bacteria from the mucus [99].

Infection of CD11c^–^CD18^+^ dendritic cells can lead to rapid entry into the systemic circulation. It has been reported by Vazquez-Torres et al. that *Salmonella* can achieve systemic dissemination through CD18-expressing phagocytes [100]. One hour after infection, *Salmonella* can be detected in the blood. At sites other than M cells and Peyer's patches, *Salmonella* can also disseminate from the gastrointestinal tract to the spleen. Downregulation of DC cells in the lamina propria can limit the invasion of *Salmonella* [100].

## 5. Macrophage

During the intracellular life of *Salmonella* in the host cells, *Salmonella* can interfere with the antigen-presenting process of the dendritic cells, for example, by interfering with the antigen presentation of bacteria on dendritic cells and inhibiting the adaptive immunity, *Salmonella* can affect the polarization of macrophages to the M2 phenotype, which will inhibit the inflammatory process and facilitate the persistent survival of *Salmonella* in the host. The manipulation of the macrophage is a strategy that *Salmonella* derived during its evolution. Uchiya et al. demonstrated that *Salmonella* can interfere with the function of macrophages to escape immune responses. Uchiya et al. reported that *Salmonella* can inhibit cytokine signaling in macrophages via the Janus kinase/signal transducer and activator of transcription (JAK/STAT) signaling pathway through SPI2 [101].

In addition to dendritic cells and M cells, *S*. typhimurium can also disseminate via inflammatory monocytes. Monocytes are recruited to the inflammatory sites where they differentiate into macrophages. Macrophages serve as a reservoir in which *Salmonella* can survive and replicate [102]. Inside the macrophage, *Salmonella* can induce micropinocytosis [103], and spacious phagosomes (SP) are formed after *Salmonella* enters the macrophage and persists in the cytoplasm [103]. A T3SS encoded by SPI2 allows survival and avoids the NADPH oxidase-dependent killing of macrophages [104].

The PhoQ/PhoP regulatory system is utilized by *S*. typhimurium to enable survival in macrophages [105]. The PhoQ/PhoP two-component system is one of the most important regulatory mechanisms for the virulence of *Salmonella.* Inside the SCV, the low PH and low Mg^2+^environment activate the two-component PhoQ/PhoP system [106]. The gene regulating the expression of O antigen, rfb, is inhibited inside the SCV [107]. Thus, the length of O antigen is decreased under the regulation of the two-component PhoQ/PhoP system. The protease PgtE in *Salmonella* typhimurium, a homologue for Pla in *Yersinia Pestis* and OmpT in *E. coli,* is then expressed [108]. Expression of PgtE protease dissolves the extracellular matrix and facilitates the cellular dissemination of *Salmonella in vivo*. *S*. typhimurium, when released from the macrophage, can then be phagocytosed by other cells, including other macrophages [109].

*Salmonella* can modify macrophage polarization during chronic infection. Macrophages can differentiate into two groups after bacterial infection; the classically activated macrophages (M1 type) or the alternatively activated macrophages (M2 type). Cytokines are the primary determinant of macrophage polarization. The M1 type is proinflammatory and activates a Th1 immune response [110]. IFN*γ*- and LPS-induced activation of TLR4 signaling can shift the macrophage to the M1 phenotype. In contrast, the M2 type is antiinflammatory and activates the Th2 immune response [110]. The cytokine IL-4 shifts macrophages to the M2 phenotype. Usually, macrophages will exhibit M1 polarization after sensing the stimuli from bacteria or viruses. Salmonella phagocytized by the macrophage can shift the macrophage polarization state. Saliba et al. reported that macrophages harboring nongrowing *Salmonella* are prone to proinflammatory M1 polarization, but macrophages harboring growing bacteria shifted to an antiinflammatory M2-like state [111]. *S.* typhimurium preferentially lives in M2 macrophages during chronic infections [110]. Thus, *Salmonella* has mechanisms to shift the differentiation of macrophages into the M2 phenotype [110]. Intracellular glucose levels are higher in M2 macrophages, contributing to their permissiveness for the intracellular replication of *Salmonella* [112].

*S*. typhimurium persists within splenic granulomas enriched with CD11b ^+^ CD11c^+^Ly6C^+^ macrophages [4, 113]. Trung et al. previously reported that *Salmonella* can manipulate granuloma macrophage polarization towards the M2 phenotype [4]. As previously discussed, *S*. typhimurium preferentially persists in M2-reprogrammed macrophages. The bacterial effector SteE contributes to the establishment of persistent infection by downregulating tumor necrosis factor (TNF) signaling [4]. The bacteria have to develop strategies to overcome the immune response and persist chronically. *S*. typhimurium can polarize the primary macrophages to M2 polarization through the *e* SPI2 T3SS effector SteE. Macrophage M2 polarization can contribute to the systemic persistence of the bacteria [113].

Studies have shown that *Salmonella* can induce host cell death during infection [114]. Monack et al. found that caspase-1 is exploited by *Salmonella* to colonize the Peyer's patches (PPs) [115]. Systemic dissemination after an oral challenge with *Salmonella* is impaired in Casp-1^−/−^ mice. This indicates that caspase-1 is important for the systemic dissemination of *Salmonella* [115]. Caspase-1 (Casp-1), an interleukin [IL]-1*β*–converting enzymes, can induce apoptosis in mammalian cells. Caspase 1 can cleave the proinflammatory cytokines IL-1*β* and IL-18. Mice lacking Casp-1 (Casp-1^−/−^mice) showed a 1,000-fold higher lethal dose (LD50) of *S*. typhimurium in the mice than wide-type mice [115]. Casp-1^-/−^mice were colonized by lower intracellular bacteria and did not show systemic dissemination of the bacteria, reduced colonization of bacteria in the Peyer's patches (PP) and spleens [115]. It suggests that Casp-1 is necessary for the establishment of systematic infection by *S*. typhimurium in mice [38, 67, 68, 115, 116]. *Salmonella* colonization was much higher in Caspase 11 deficient mice than in wild-type mice [68]. Casp1^−/−^ and Casp1/11^−/−^ monolayers showed significantly increased intracellular bacteria, accompanied by low intestinal epithelial cells (IECs) shedding and death [68]. Caspase activation is important for limiting the intracellular replication of *Salmonella*.

Inflammasome activation is one important pathway during the infection of *Salmonella* in the intestinal epithelial cells [38]. The infection of *Salmonella* typhimurium can also lead to the activation of Caspase 4, and Caspase 4 can limit the replication of *S*. typhimurium in the cells [117]. Activation of caspase 4 can lead to the noncanonical activation of the inflammasome pathway [117].

*Salmonella* can activate apoptosis of *Salmonella*-infected macrophages using effectors encoded in pathogenicity island-1 through both intrinsic and extrinsic pathways [118]. Cell death induced by the infected cells gives the bacteria an opportunity to be released and infect further cells. *Salmonella* can induce cell death in macrophages through several mechanisms. Immediate cell death can be induced by the type III secretion system (T3SS) of *Salmonella*. Or, the macrophages harboring *Salmonella* can be further phagocytosed by neighboring macrophages. Bacteria are released from dead cells and phagocytized by local macrophages, enabling another cycle of intracellular replication and cell-to-cell spread [114]. Ultimately, this cycle helps ensure the intracellular survival and persistent infection of phagocyte populations with *Salmonella*.

## 6. Chronic and Systemic Infection of *Salmonella* Typhimurium

Supershedders are the hosts responsible for the host-to-host transmission and reoccurrence of S. typhimurium since supershedders shed the bacteria in their feces. Foxp3þ Regulatory T cells play a role in the persistent infection of *Salmonella* [119]. Foxp3^+^ Treg ablation early after infection will accelerate bacterial eradication [119]. This indicated that immune regulatory T cells function in the early stages of infection to establish a persistent *Salmonella* infection [119].

Monack et al. demonstrated that *Salmonella* can persist in the MLNs of mice for up to one year. Macrophages in the MLNs can be the reservoirs of the bacteria. Voedisch et al. suggested that the MLN represents a restrictive site for the growth and dissemination of *Salmonella* [33]. In mice whose mesenteric lymph nodes have been surgically excised, the colonization of *Salmonella* in the liver and spleen is increased [33]. In such mice, *Salmonella* forms nonreplicating “persisters” in macrophages [120]. Persisters are in a state of dormant infection that is tolerant to drug treatment [121]. Indeed, they have resistance to antibiotics and can eventually reactivate and begin to replicate once more [122]. Persister cells are one important reason for relapsed infections. Persisters facilitate the chronic infection with *S*. typhimurium. Persisters can undermine the host immune response [123]. These persisters can reprogram the macrophages they dominate [123]. After exposure to ciprofloxacin, a fluoroquinolone antibiotic, *Salmonella* enterica persisters form unstable small colony variants. These phenotypes help the bacteria survive in the face of environmental stress or antibiotic treatments.

*Salmonella* persister cells are important components of biofilms [124]. Biofilm formation is an important strategy for persistent bacterial infections [125]. Forming biofilm can confer the bacteria survival advantages. Biofilm formation on gallstones is important for the chronic carriage of *Salmonella*. Antibiotic therapy efficiency is compromised in patients with a biofilm in the gall bladder. *Salmonella* infection in the gall bladder can induce the destruction of the epithelial cell integrity.

Biofilms are just one strategy for the bacteria to survive harsh environments. Even without animal reservoirs, biofilms can help *Salmonella* spp. to survive in the environment until uptake into a new host. However, the *Salmonella* Typhimurium ST313 strain which can cause blood stream infections and is typically seen in Sub Saharan Africa [126], has poor biofilm-forming ability and cannot survive long outside a host [127].

Except in antigen-presenting cells, *Salmonella* achieves a persistent infection in epithelial cells [128] by remaining in a dormant state. Luk et al. found that *Salmonella* can live in a dormant state in the vesicular compartment, different from the *Salmonella*-containing vacuoles (SCV). Contrary to macrophages, *Salmonella* in epithelial cells can express *Salmonella* Pathogenicity Island 2 (SPI-2) virulence factors. This report is the first to describe another persistent infection state and mechanism for S. typhimurium [128].

The *Salmonella* SPI2 effector SseI (also called SrfH) binds with host factor IQ motifs containing GTPase activating protein 1 (IQGAP1). SseI has been reported to mediate long-term systemic infections [60]. Pseudogenization of SseI leads to rapid systemic dissemination of *Salmonella* typhimurium through migratory dendritic cells [129]. In the sub-Saharan African *Salmonella* typhimurium strain ST313 lineage II, sseI is lost by pseudogenization. ST313 can disseminate from the gut to mesenteric lymph nodes (MLNs) via CD11b + migratory dendritic cells (DCs) [129]. However, recovery of the gene function by expressing functional SseI in ST313 isolates reduces the dissemination of the bacteria [129].

The interplay between the host immune system and pathogens is a complex process during chronic infections. Dendritic cells and macrophages are important reservoirs for the bacteria that enable long-term survival. *Helicobacter pylori*, *Mycobacterium tuberculosis*, and *Salmonella* enterica all survive inside antigen-presenting cells (APCs). The gall bladder, bone marrow [130], and mesenteric lymph nodes are sites that can support persistent infection with *Salmonella*. Persistent infection with *Salmonella* can cause disease in multiple organs, from gallbladder cancer to pancreatitis. Pancreatitis can be caused by persistent infection of mice with *Salmonella* [14]. Inflammatory, fibrotic, and epithelial responses can be detected in the pancreases of mice persistently infected with S. typhimurium [14]. Pancreatic acinar cells can be invaded by S. typhimurium.

*Salmonella* infections are associated with the development of IBD (inflammatory bowel diseases) and colon cancer [131, 132]. One study by Katrin et al. reported that mice with chronic infections with *S.* typhimurium develop severe and persistent intestinal fibrosis and have upregulation of several matrix metalloproteinases (MMPs) [133]. Transforming growth factor–*β*1, insulin-like growth factor-I, and type I collagen deposition levels are increased during persistent infection of *S*. typhimurium [134, 135].

As shown in mouse models, chronic infection with *S*. typhimurium increases the susceptibility to intestinal inflammation [136]. The dDextran sulfate sodium (DSS)-induced colitis and interleukin (IL)-10^−/−^ spontaneous inflammation mice models were used in this particular study [137]. Because of persistent infection of *S*. typhimurium in the liver and spleen, these mice are more susceptible to intestinal inflammation. This indicated *S*. typhimurium persistent infection might be related to the accelerated onset of IBD (inflammatory bowel diseases) of the host [137].

Various studies support the mesenteric lymph nodes as a site that harbors *Salmonella* to sustain a chronic infection [138]. *Salmonella* can persist in the hemophagocytic macrophages of MLN. Removal of MLN increases the bacterial burdens in mice, however, indicating that another reservoir of *Salmonella* exists other than MLN [138]. Bacteria can be cultured from the liver tissue of chronically infected mice [139]. Liver macrophages are shifted to the M2 phenotype during persistent infection. An immune response balance exists during chronic infection with *Salmonella*, for example, the proinflammatory IFN*γ* and antiinflammatory signals IL-10. This balance allows the bacteria to survive in the persistent infection sites [139].

The cytokine Interleukin-22 (IL-22) can help the colonization of *Salmonella* by suppressing other commensal bacteria [140]. IL-22 can function in tissue repair and host defense; it is induced during pathogen infection. Behnsen et al. reported that IL-22 can suppress the intestinal microbiota [140]. IL-22 suppresses commensal *Enterobacteriaceae* and boosts the colonization of *Salmonella*. Binding of bacteria with APCs will induce the release of cytokine IL-23; IL-23 induces IL-17 and IL-22 release [141, 142]. In IL-22^−/−^ mice has higher *E. coli* burden and reduced *Salmonella* colonization in the intestine than wide type mice. IL-22 can induce the antimicrobial proteins lipocalin-2 and calprotectin release to inhibit the growth of commensal microbiota. This mechanism is exploited by *Salmonella* to outcompete intestinal microbiota [140].

## 7. Concluding Remarks

Achieving a better understanding of the pathogenesis of *Salmonella* will provide further insights into key host-pathogen interactions that affect persistent bacterial infections. Understanding the detailed mechanisms and the specific host cell types involved in *Salmonella* infections may help guide the future development of therapeutic interventions. Understanding the mechanisms of *Salmonella* persistent infection will enable researchers to improve upon current treatment strategies, especially for asymptomatically infected patients. Treating chronically infected patients will help reduce the reservoirs for the bacteria and limit the transmission of the disease.

## Figures and Tables

**Table 1:** Effectors of Type III secretion systems in the invasion and dissemination of *Salmonella*.

Type III secretion System	Function	References
SPI-1	Invasion of nonphagocytic cells, including epithelial cells	[40]
SPI-2	Survive in the phagocytic cells	[59]
SseI	Inhibition of macrophages and DCs migration	[60]
SipA	Promotion of cytoskeletal rearrangements, invasion of epithelial cells	[61, 62]
SopA	Invasion of epithelial cells	[63]
SopB	Invasion of epithelial cells	[63]
SopD	Invasion of epithelial cells	[63]
SopE2	Invasion of epithelial cells	[63]
SipB/C	Translocator to deliver the effectors into the cell	[49]
